# Intrapartum Intrauterine Fetal Demise with Normal Umbilical Cord Blood Gas Values at Birth

**DOI:** 10.1155/2015/318350

**Published:** 2015-01-19

**Authors:** Michael D. Benson

**Affiliations:** Department of Obstetrics and Gynecology, Feinberg School of Medicine, Northwestern University, 101 Bentley Court, Deerfield, IL 60015, USA

## Abstract

A case is presented in which a fetus was delivered by cesarean section for failure to progress and a “nonreassuring heart rate tracing” in which the Apgar scores were unexpectedly 0 at 1, 5, and 10 minutes. Resuscitation was unsuccessful after 30 minutes. The venous cord gas was normal and the arterial blood gas was not consistent with intrapartum asphyxia. At the time of surgery, the placenta appeared grossly normal. The autopsy was entirely normal. This case raises questions about our understanding of intrauterine fetal demise and suggests an approach to future research.

## 1. Introduction

Intrauterine fetal demise (IUFD) occurs in roughly 6 per thousand pregnancies [1]. Of 205 stillbirths in the third trimester at one institution over 10 years, 12 occurred during labor [2]. Common causes of fetal death include congenital malformations, intrauterine infection, and sudden, catastrophic intrapartum events. A case is presented in which an unexpected IUFD occurred in the presence of normal umbilical cord gas profile at birth (see Table 1).

## 2. Case Presentation

A 34-year-old G1 P0 at 36 0/7 weeks was admitted at 11:55 with a complaint of spontaneous rupture of membranes. Examination revealed abundant clear fluid leaking from the vagina, a cervical exam of 4 cm, complete effacement and 0 station, and a Category I fetal heart rate tracing. Oxytocin augmentation of labor was begun shortly after admission, and the patient received epidural anesthesia at 18:25. At 19:55, an 8-minute prolonged deceleration to 60 BPM occurred which then recovered to a baseline of 140 with moderate variability and presence of accelerations. The fetal heart rate tracing returned to Category I (baseline of 140, presence of moderate variability and accelerations, and absence of decelerations) until 23:30 at which point recurrent variable decelerations occurred with moderate variability for the remainder of the tracing [3]. Her cervix was fully dilated and the presenting part was 1 cm below the level of the ischial spines at 0005, and she began pushing. At 02:37, the patient had a second prolonged deceleration lasting six minutes and reached a nadir of 60 BPM for approximately one minute. It recovered to a baseline of 150 and return of accelerations and moderate variability. Exam at that time revealed no descent and continued right occiput posterior position.

A decision was made for cesarean section due to arrest of descent and “nonreassuring fetal heart rate status” at 02:48. Continuous monitoring ceased at 02:58 for the move to the operating room. Fetal heart tones in the operating room at 03:10 were obtained briefly at the rate of 115 BPM. The maternal pulse at that time was 82. The skin incision was begun at 03:17, and the uterine incision occurred at 03:25. As the fetal head was firm-lodged in the maternal pelvis, a number of maneuvers were required to accomplish delivery including an attempt to dislodge the head from below by an assistant and the summoning of a second obstetrician to the operating room for further assistance. There was no evidence of physical cord compression at the time of delivery. A 2,934-gram male with Apgar scores of 0/0/0 was delivered at 03:32. Resuscitation, including intubation, epinephrine, placement of a UVC line, and fluid resuscitation, failed to elicit any signs of life and was discontinued after 30 minutes.

Autopsy revealed an anatomically normal male. Although placental histology was not obtained, the placenta was described as “normal” upon its delivery by the obstetrician. Mother was discharged without further complications on postoperative day 2. The patient's antepartum and postpartum leukocyte counts were normal; she was afebrile throughout her hospital course.

## 3. Discussion

This case describes an unexpected stillborn delivered at the time of cesarean section for arrest of descent (two hours in the second stage). Although, “nonreassuring fetal heart rate tracing” was an additional diagnosis, the Category II tracing was not predictive of acidosis and had moderate variability, presence of accelerations, and a baseline of 140 throughout. The arterial and venous blood gas values were not consistent with acidosis. This case came to the reviewer's attention on behalf of counsel for the patient who was seeking a medical-legal review. No standard-of-care violations were found. Subsequently, the patient gave permission for these events to be written up as a case report for the medical literature.

The diagnostic work-up was admittedly less than what might be desired. No fetal CBC, serology, fetal karyotype, or placental pathology was obtained. The hospital does not routinely report specific values for partial pressures of oxygen <25 mmHg and does not calculate base deficit. Using a blood gas nomogram, the umbilical artery base excess is calculated to be −10 (assuming a fetal hemoglobin at birth of 15 gm/dL) [4].

The 18 minutes from procedure “start” to birth and uterine incision to birth were both longer than average. In one study of over 200 cesarean sections, 17% of skin incision-to-birth intervals were longer than 18 minutes while 35% of cesareans had a uterine incision-to-birth interval longer than 7 minutes [5]. The authors found no relationship between both time interval and newborn outcome, a finding confirmed in a second study of over 900 cesareans [6]. With normal cord gases, no evidence of external or internal trauma, and a normal autopsy, no theory presented itself as to how a longer than average uterine incision-to-birth interval or how the extra delivery maneuvers could have resulted in the fetal demise.

One obvious etiology of fetal demise in this case would have been an unrecognized fetal-maternal transfusion or fetal hemorrhage. Unfortunately, neither a hemoglobin-acid elution test nor a newborn hemoglobin test was obtained, so this remains a possibility. However, the heart rate tracing near delivery did not show a pattern characteristic of fetal anemia severe enough to cause death and there was no apparent trauma to either the fetus or the placenta. As a result, a fetal hemorrhage seems unlikely.

Another type of etiology that might have benefited from greater investigation was genetic abnormalities. Although the baby was phenotypically normal, a karyotype was not done. Perhaps, more importantly, new research is finding lethal genetic errors at the subchromosome level, microdeletions [7]. Although no genetic tests were done here, these abnormalities remain an unlikely explanation for this newborn's sudden demise just before birth.

It is possible that a prolonged bradycardia occurred as a result of redosing of the epidural, as reported previously in the literature [8]. However, there were no corresponding drops in maternal blood pressure, making this mechanism less likely. The lack of observed maternal hypotension made aortocaval compression with concomitant decrease in uterine blood flow also unlikely. Finally, the possibility remains that pressure on the baby's face in an effort to dislodge the head might have induced a vagally mediated bradycardia which then led to asystole. However, this mechanism has not previously been reported.

Although most authorities would regard the cord gasses as normal, a pH difference of >0.10 between arterial and venous values has been described as widened [3]. This finding has been ascribed to a circumstance where the lower pressure umbilical vein is occluded, while the higher pressure umbilical artery is not. While, in fact, the fetal heart tracing did exhibit variable decelerations, which are consistent with cord occlusion, nothing in the tracing was suggestive of fetal acidosis. Also, while there might have been a widened gap between the pHs, in point of fact, both of the arterial and venous umbilical cord gases were normal. An alternative explanation for a “widened” difference between cord gasses has been ascribed to chronic heart failure, for which there is no evidence here [3].

Finally, a sudden, fatal arrhythmia has been suggested as a possible cause of a dead newborn with normal blood gasses [3]. In fact, another such case report of a dead fetus with normal arterial and venous gases was described in a 1987 case report in* Obstetrics and Gynecology* [9]. In an effort to explain this, asystole was induced in fetal lambs, and the authors found that arterial cord gas values did not change much for at least ten minutes after asystole. As a result, they concluded that fetal asystole might be a reason for these uncommon cases of dead neonates with normal cord gasses at birth.

A literature review using a variety of search term combinations failed to identify any other reports of normal umbilical cord gasses and very low Apgar scores (i.e., 1-minute score of 0 or 1) other than the one case described here. The author is personally aware of three other cases in which the one-minute Apgar score was either 0 or 1 in the presence of normal cord gasses and no fetal-maternal transfusion. Two of these three other newborns succumbed.

It is worth emphasizing that there can be no doubt that dead newborns with normal blood gasses likely experienced asystole within minutes of birth. It is one thing to explain, how a dead baby can have normal cord gasses after death, and certainly sudden asystole close to birth makes sense. However, the issue in this case and the others like it is what caused the asystole in the first place?

One intriguing explanation that has not been explored up to now is a sudden, sustained drop in fetal blood pressure—before a cardiac arrhythmia or asystole. As yet, unidentified inflammatory pathways could mediate such hypotension. This is a specific, testable hypothesis. This mechanism can be evaluated through proteomic assays for cytokines in the umbilical cord blood of these occasional newborns with Apgar scores of zero and normal cord gasses. The NIH's Maternal Fetal-Medicine Units Network has already published studies on stillbirths. Perhaps this small subset intrauterine fetal demise immediately before birth could be studied further to gain insight into fetal physiology.

Anatomically normal newborns at term with normal cord gasses but with very depressed Apgar scores are uncommon. This case suggests that they do exist and merit further study. Potential etiologies could include sudden fetal hypotension, perhaps mediated by inflammation.

## Figures and Tables

**Table 1:** Umbilical cord gases^*^.

	Venous	Arterial
pH	7.27	7.13
pCO_2_	48	64
pO_2_	<25	<25
Bicarbonate	21.6	20.7
Base excess^*^	−10	−5

^*^Base excess/deficit calculated using nomogram found in Pomerance [3].
